# Energy state of InGaAs quantum dots on SiO_2_-patterned vicinal substrate

**DOI:** 10.1186/1556-276X-7-104

**Published:** 2012-02-06

**Authors:** Hyo Jin Kim, Junichi Mothohisa, Takashi Fukui

**Affiliations:** 1 Photonic Energy Research Center, Korea Photonics Technology Institute, Wolchul dong 971-35, Buk-gu, Gwangju, Korea; 2 Research Center for Integrated Quantum Electronics, Hokkaido University, North 13 West 8, Sapporo 060-8628, Japan

## Abstract

The optical properties of In_0.8_Ga_0.2_As self-assembled quantum dots (SAQDs) grown on GaAs wire structures formed by utilizing SiO_2_-patterned exact and 5°-off (001) GaAs substrates have been studied with micro-photoluminescence (μ-PL). Single PL peak was occurred for In_0.8_Ga_0.2_As SAQDs grown on SiO_2_-patterned exact (001) GaAs, whereas double PL peaks were showed for SAQDs grown on 5°-off (001) GaAs substrates as the width of the opening windows increased. The power-dependent μ-PL spectra show that the first and second peaks of these double peaks were originated from the well-defined ground and excited state, respectively. These results demonstrated that In_0.8_Ga_0.2_As SAQDs selectively grown by utilizing SiO_2_-patterned 5°-off (001) GaAs substrates have well-defined zero-dimensional quantum states.

## Introduction

Self-assembled quantum dots (SAQDs) which can be formed by Stranski-Krastanow growth mode have been demonstrated to be defect free and to have high density with three-dimensional quantum confined nature of the electronic spectra. However, the randomness in their size as well as position on a planar substrate is undesirable particularly for electronic device applications, even though they produce these unique properties for the realization of quantum functional electron devices [1,2]. For this reason, many techniques have been proposed and attempted to control the spatial distribution, such as growth on miscut substrates with surface steps, growth on relaxed templates with dislocation network, stacking growth of multi-layers of islands, and so on [3-5].

Among them, selective area metalorganic vapor phase epitaxy (SA-MOVPE) is one of the most effective approaches in fabrication of uniform and position-controlled QDs because appropriate patterning of the mask layer and control of the growth conditions enable us to realize their control without any fabrication damage introduction into the epitaxial layers. However, the selective area growth (SAG) of SAQDs on patterned exact (001) GaAs substrates has difficulties for the control of the interval or position of multiple SAQDs, which are also important for quantum electronic device.

We reported previously that In_0.8_Ga_0.2_As SAQDs having regular periodicity on a narrow (001) top terrace of GaAs layer formed on a SiO_2_-stripe patterned 5°-off (001) GaAs substrate [5,6]. The bunching effect of the GaAs layer along the misorientation direction (M_||_) on (001) top facet was maintained using a substrate having a high misorientation angle, so that In_0.8_Ga_0.2_As SAQDs selectively grown on multi-atomic step on (001) top facet of GaAs layer. This new technique was the first trial to control multiple SAQDs by a combination of miscut substrate and SAG method.

In this letter, we investigated that the optical properties of In_0.8_Ga_0.2_As SAQDs on GaAs wires were formed by utilizing SiO_2_-patterned exact and 5°-off (001) GaAs substrates.

The energy states and formations of SAQDs were represented by the experimental results of micro-photoluminescence (μ-PL) and scanning electron microscope (SEM), respectively. The discrete natures of zero-dimensional density of state in In_0.8_Ga_0.2_As SAQDs were dramatically changed as the misorientation angles of substrates and (001) top facet width of GaAs wires (W_(001)_) were varied. Also, the ground and excite states of SAQDs with various SiO_2_-pattterns were investigated using the power-dependent μ-PL.

## Experiment

Starting materials used in this study were exact and 5°-off (001) GaAs with patterned SiO_2 _as a mask. The direction of misorientation angles is $\left[1\bar{1}0\right]$ and the thickness of SiO_2 _is 20 nm. The whole patterns consisted of different 25 kinds of pattern regions which were filled with stripe windows (wire region) along $\left[1\bar{1}0\right]$ direction in 800 nm-periodicity. Twenty-five kinds of patterns have different widths of opening region (W_0_) which were varied from 300 to 700 nm. The detailed pattern shapes were represented in our previous study [5]. The growth of GaAs buffer layer and In_0.8_Ga_0.2_As SAQDs were performed by low-pressure MOVPE (LP-MOVPE) working at 76 Torr. The growth temperature for the GaAs and In_0.8_Ga_0.2_As SAQDs was 700 and 500°C, and the corresponding nominal thicknesses were 200 nm and 3.2 ML, respectively. The growth thickness and temperature of GaAs cap layer for PL were 100 nm and 500°C, respectively. The structural and optical properties were investigated by SEM and μ-PL. μ-PL measurement were carried out with a ×100 microscope objective which can focused the excitation beam into < 3 μm diameter spot on the samples mounted in a variable temperature cold-finger cryostat. 633 nm He-Ne laser in continuous-wave (cw) operation was used as excitation source. During this experiment, care was taken to achieve high positional stability and reproducibility of the expectation position on the sample at 4 K.

## Results and discussion

**Figure 1:**
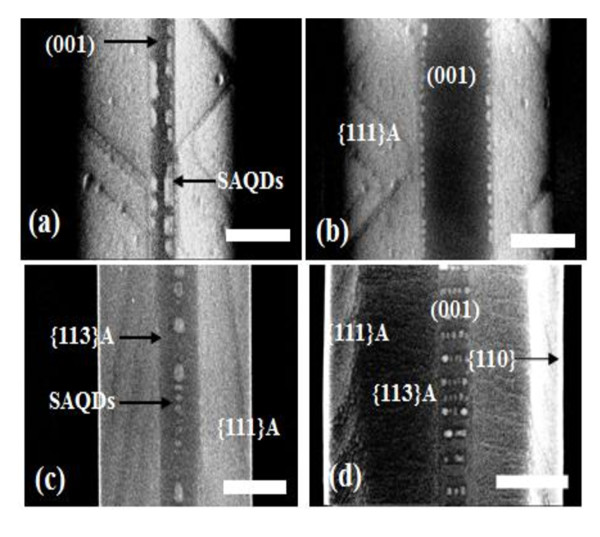
**SEM images of In_0.8_Ga_0.2_As SAQDs on GaAs buffer layers were formed on SiO_2_-patterned exact (001) GaAs substrate with W_0 _of (a) 275 and (b) 630 nm, respectively**. SEM images of In_0.8_Ga_0.2_As SAQDs on GaAs buffer layers were formed on SiO_2_-patterned 5°-off (001) with W_0 _of **(c) **285 and **(d) **730 nm, respectively.

Figure 1a, b shows that SEM images of In_0.8_Ga_0.2_As SAQDs on GaAs buffer layers were formed on SiO_2_-patterned exact (001) GaAs substrate with W_0 _of 275 and 630 nm, respectively. During the growth of the GaAs buffer layer on opening region of SiO_2_-patterned exact (001) GaAs substrates, the formations of GaAs buffer layers have been changed to mesa-structure which consist of (001) top facet and {111}A facets on side walls. The widths of the (001) top facet (W_(001)_) were directly proportional to W_0 _and the corresponding W_(001) _were 57 and 185 nm, respectively. In_0.8_Ga_0.2_As SAQDs were not formed on {111}A facet, and grew selectively on the edge region of (001) top facet as shown in Figure 1a, b. This is because the growth rate of edge region on (001) facet was relatively increased by the surface migration of In adatoms from the {111}A sidewalls to the (001) top facet (M_S→T_) with the proper growth thickness of In_0.8_Ga_0.2_As layer [7-9].

Figure 1c, d shows that SEM images of In_0.8_Ga_0.2_As SAQDs on GaAs buffer layers were formed on SiO_2_-patterned 5°-off (001) GaAs substrate with W_0 _of 285 and 730 nm, respectively. The corresponding W_{113}A _and W_(001) _were 52 and 0, and 214 and 724 nm, respectively. As W_(001) _was increased, the interval SAQDs on GaAs wire was clearly showed. This is because the surface migrations of Ga atoms from (111) side wall to (001) top facet were decreased relatively, so that the bunching effects for multi-atomic step were maintained. Also, one of the different properties of GaAs grown on SiO_2 _patterned 5°-off (001) GaAs substrate was the appearance of the surface {110} and {113}A. Figure 1c, d shows that the widths of {111}A (W_{111}A_), {113}A (W_{113}A_) were narrower and wider as the W_0 _and W_(001) _was increased. For the GaAs wires grown on SiO_2 _patterned 5°-off (001) GaAs, both {113}A and {111}A facets include steps perpendicular to the misorientation direction for the misorientation substrates. Average distance between steps is estimated to be 3.2 nm for 5°-off (001) GaAs substrate. These steps probably work as nucleation sites for surface migrating Ga atoms, and thus enhance growth rate [10]. The appearance of the wider W_{113}A_, vertical W_{110}_, and the narrower W_{111} _with wider W_0 _indicates that the growth rates of {111}A are not so lower than (001) top facet. This effect allows stable bunching effect of multi-atomic step on (001), and SAQDs selectively formed on step edge with definite interval as shown in Figure 1d.

**Figure 2:**
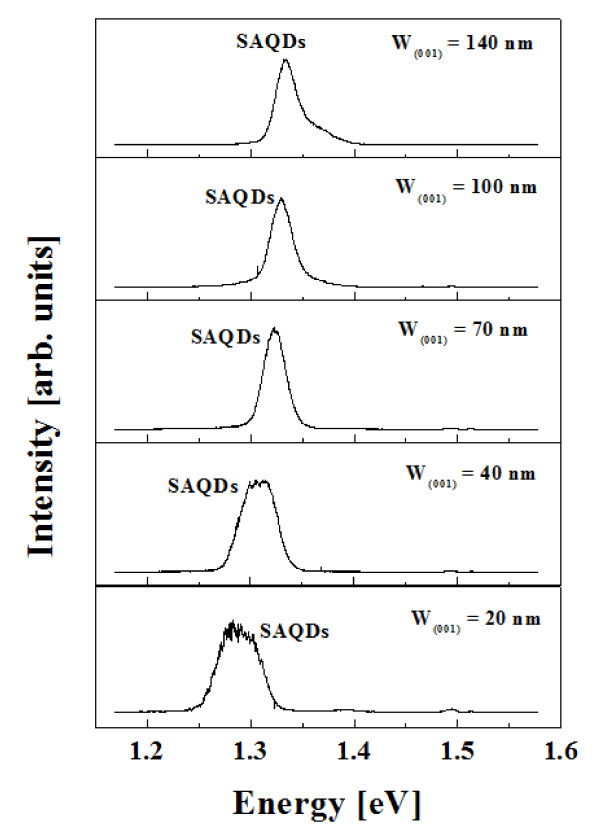
**μ-PL spectra at 4.0 K of the In_0.8_Ga_0.2_As SAQDs ensemble grown on SiO_2_-patterned exact (001) GaAs substrates with W_(001) _of (a) 20, (b) 40, (c) 70, (d) 100, and (e) 140 nm, respectively**.

Figure 2a, b, c, d, e shows the μ-PL spectra at 4.0 K of the In_0.8_Ga_0.2_As SAQDs ensemble grown on SiO_2_-patterned exact (001) GaAs substrates with W_(001) _of 20, 40, 70, and 100, and 140 nm, respectively. As shown in Figure 2a, b, c, d, e, the energy states of SAQDs had single peak after (001) top region appeared. The full width half maximum (FWHM) of energy peaks was broad until W_(001) _was 70 nm, after which FWHM maintained to 30 meV with stable state. As W_(001) _increased, the size of SAQDs was smaller with the blue shift of energy state of SAQDs. As W_(001) _were 20, 40, 70, 100, and 140 nm, the energy states of SAQDs were 1.29, 1.30, 1.32, 1.33, and 1.33 eV, respectively. The energy states of SAQDs for the wetting layer were not occurred.

**Figure 3:**
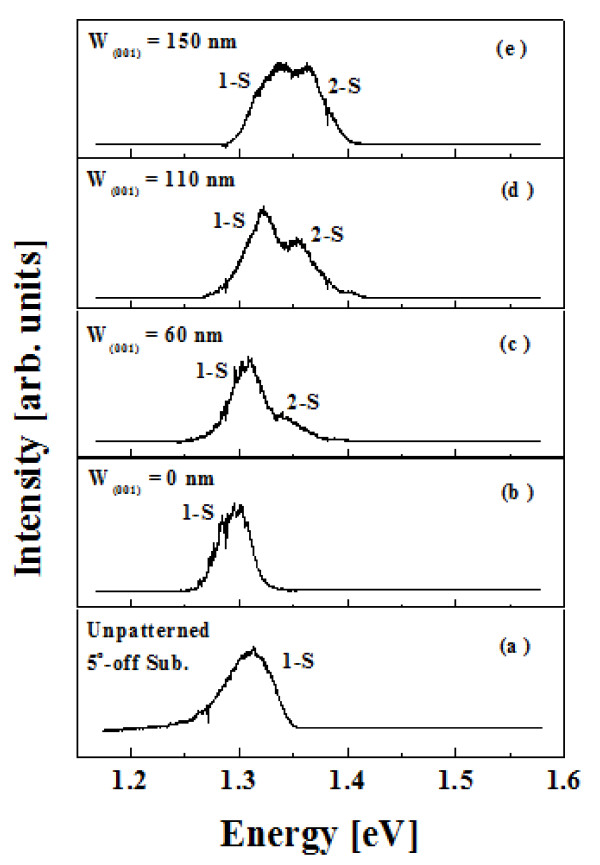
**(a) The μ-PL spectrum of In_0.8_Ga_0.2_As SAQDs grown on unpatterned 5°-off (001) GaAs substrates and μ-PL spectra at 4.0 K of the In_0.8_Ga_0.2_As SAQDs ensemble grown on SiO_2_-patterned 5°-off (001) GaAs substrates with W_(001) _of (b) 0, (c) 60, (d) 110, and (e) 150 nm, respectively**.

Figure 3b, c, d, e shows the μ-PL spectra at 4.0 K of the In_0.8_Ga_0.2_As SAQDs ensemble grown on SiO_2_-patterned 5°-off (001) GaAs substrates with W_(001) _of 0, 60, 110, and 150 nm, respectively. The reason for the existence of the single peak with W_(001) _of 0 means the formation of SAQDs with broad FWHM as shown in Figure 1c. Figure 3b, c, d, e shows that the double peaks were occurred as (001) top area appeared. The single peak of SAQDs with large fluctuation was occurred up to reach a certain value of W_(001)_, after which the second peak more increased as W_(001) _was wider. Also the energy state of the first and second peaks had slightly blue shift. As the W_(001) _were 60, 110, and 150 nm, the corresponding energy states of first peaks were 1.31, 1.32, and 1.33 eV, respectively. The corresponding energy states of the second peaks were 1.33, 1.35, and 1.36 eV, respectively. On the other hand, the different tendency for unpatterned 5°-off (001) GaAs substrate was found. Figure 3a shows the μ-PL spectrum of In_0.8_Ga_0.2_As SAQDs grown on unpatterned 5°-off (001) GaAs substrates. The single peak with the energy state of 1.31 eV was occurred.

In order to investigate the origin of first and second peaks, we have investigated the dependences of the intensities for two peaks of SAQDs on the excitation power of laser.

**Figure 4:**
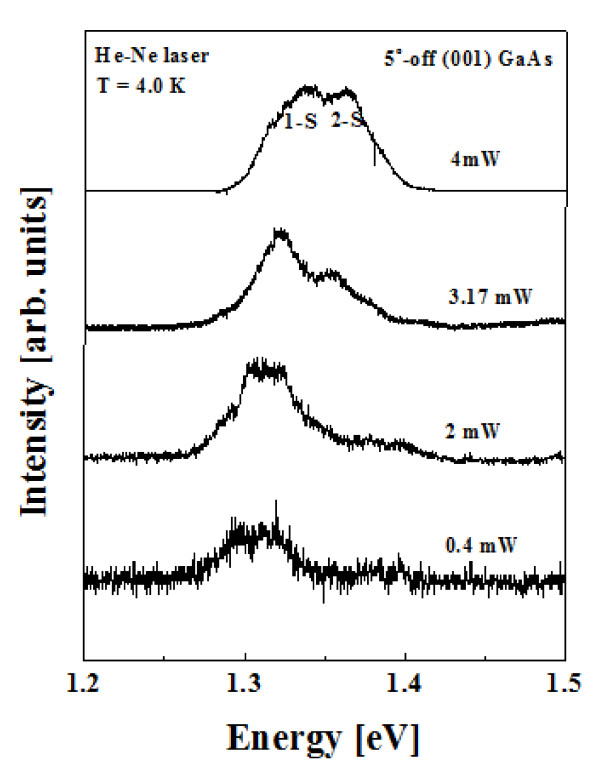
**μ-PL spectra of an In_0.8_Ga_0.2_As SAQDs ensemble grown on SiO_2_-patterned 5°-off (001) GaAs substrate with the excitation power of He-Ne laser were (a) 0.4, (b) 2, (c) 3.17, and (d) 4 meV, respectively**. (W_(001)_:150 nm).

Figure 4a, b, c, d shows that μ-PL spectra of an In_0.8_Ga_0.2_As SAQDs ensemble grown on SiO_2_-patterned 5°-off (001) GaAs substrate with the excitation power of He-Ne laser were 0.4, 2, 3.17, and 4 meV, respectively, when W_(001) _was maintained as the constant value of 150 nm. As shown in Figure 4a, b, c, d, the intensities of the first peaks saturated as the excitation power of laser increased, whereas the intensities of the second peaks increased. According to the previous study for InAs QDs formed on GaAs pyramids [11], the experimental results for the dependence of the ground and the excited states of PL peaks on laser power had the similar tendency as in our results. Also, the difference of the energy states for double peaks was about 0.3 eV as same as the value in our results. These results indicate that the first and second peaks are attributable to the filling of the ground state and the resultant recombination from excited states as the power of laser increased [11]. Also, these results directly demonstrate the discrete natures of the densities of states in In_0.8_Ga_0.2_As SAQDs which are selectively grown on SiO_2_-patterned 5°-off (001) GaAs substrate as the W_(001) _was wider as shown in Figure 3b, c, d, e. The reason for the difference of the optical properties of SAQDs formed on GaAs wire grown by using SiO_2_-patterned exact (001) and 5°-off (001) GaAs substrates was related to the dissimilar surface migration of Ga adatoms from sidewall to (001) top region.

**Figure 5:**
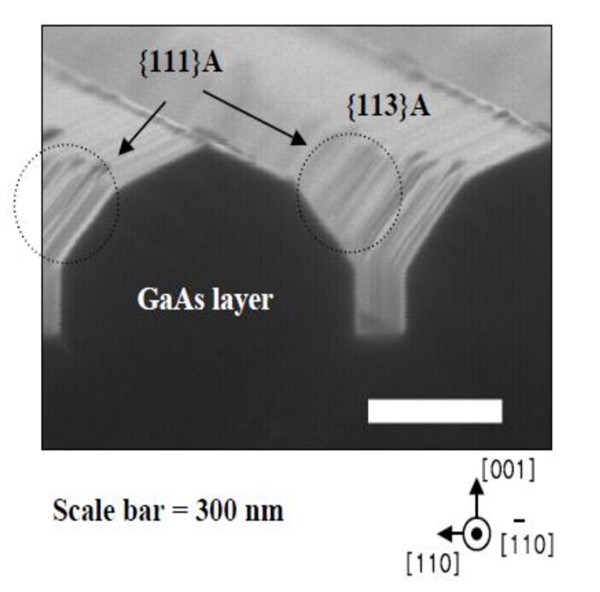
**Cross sectional SEM image of the GaAs layer on SiO_2_-patterned 5°-off (001) GaAs substrate, when W_0 _was 655 nm**. The front direction of GaAs layer was rotated about 30° from $\left[1\bar{1}0\right]$ direction.

Figure 5 shows the cross section of GaAs wires grown by utilizing SiO_2_-patterned 5°-off (001) GaAs substrate observed from 30° tilted $\left[1\bar{1}0\right]$ direction. The W_0 _of this sample was 655 nm. Corrugations caused by the step bunching were clearly observed on {111}A facets. This result indicates that the growth rate on the {111}A facets was enhanced by the high step density because the step bunching effect of the GaAs was occurred by the high incorporation rates of Ga atoms at multi-atomic steps on the {111}A facets. Therefore, we believe that the excellent formations of SAQDs having well-confined quantum nature on (001) top terrace are caused by the nucleation site because of multi-atomic step and smaller surface migration of In and Ga adatoms from sidewalls using 5°-off (001) GaAs and the wider (001) top terrace width.

## Summary

We investigated that the energy states of In_0.8_Ga_0.2_As SAQDs on GaAs wires were formed by utilizing SiO_2_-patterned exact and 5°-off (001) GaAs substrates. In_0.8_Ga_0.2_As SAQDs by utilizing SiO_2_-patterned GaAs were grown selectively on the edge region of (001) top facet of GaAs wires, whereas those by utilizing 5°-off (001) GaAs were formed with the periodicity on the multi-atomic steps on (001) top terrace of GaAs wires with wider W_(001)_. The optical properties of SAQDs were investigated by μ-PL spectra at 4.0 K. Single peak for the energy state of SAQDs by utilizing SiO_2_-patterned exact (001) was showed. On the other hand, double peak was occurred for SiO_2_-patterned 5°-off (001) GaAs substrate. According to the experimental results of μ-PL spectra on the various laser powers, the intensity of the first peak saturated as the excitation power of laser increased, whereas the intensity of the second peak increased. These results indicate that the energy states of first and second peaks mean the ground and excited states, respectively. Therefore, it was confirmed that the excellent formation of SAQDs having well-confined quantum nature could be obtained by utilizing SiO_2_-patterned 5°-off (001) GaAs substrates.

## Competing interests

The authors declare that they have no competing interests.

## Authors' contributions

The work presented here was carried out in collaboration among all authors. Professor TF and JM help HJK to carry out the laboratory experiments and analyzed the data. All authors read and approved the final manuscripts.
